# Sustainable Starch/Lignin Nanoparticle Composites Biofilms for Food Packaging Applications

**DOI:** 10.3390/polym15081959

**Published:** 2023-04-20

**Authors:** Xunwen Sun, Qingye Li, Hejun Wu, Zehang Zhou, Shiyi Feng, Pengcheng Deng, Huawei Zou, Dong Tian, Canhui Lu

**Affiliations:** 1 State Key Laboratory of Polymer Materials Engineering, Polymer Research Institute, Sichuan University, Chengdu 610065, China; 2 Jiangsu Key Laboratory for Carbon-Based Functional Materials & Devices, Institute of Functional Nano & Soft Materials (FUNSOM), Soochow University, Suzhou 215123, China; 3 College of Science, Sichuan Agricultural University, Ya’an 625014, China; 4 Institute of Ecological and Environmental Sciences, Sichuan Agricultural University, Chengdu 611130, China; 5 Advanced Polymer Materials Research Center, Sichuan University, Shishi 362700, China

**Keywords:** lignin nanoparticles, starch composite films, antioxidant, UV shielding, packaging

## Abstract

Construction of sustainable composite biofilms from natural biopolymers are greatly promising for advanced packaging applications due to their biodegradable, biocompatible, and renewable properties. In this work, sustainable advanced food packaging films are developed by incorporating lignin nanoparticles (LNPs) as green nanofillers to starch films. This seamless combination of bio-nanofiller with biopolymer matrix is enabled by the uniform size of nanofillers and the strong interfacial hydrogen bonding. As a result, the as-prepared biocomposites exhibit enhanced mechanical properties, thermal stability, and antioxidant activity. Moreover, they also present outstanding ultraviolet (UV) irradiation shielding performance. As a proof of concept in the application of food packaging, we evaluate the effect of composite films on delaying oxidative deterioration of soybean oil. The results indicate our composite film could significantly decrease peroxide value (POV), saponification value (SV), and acid value (AV) to delay oxidation of soybean oil during storage. Overall, this work provides a simple and effective method for the preparation of starch-based films with enhanced antioxidant and barrier properties for advanced food packaging applications.

## 1. Introduction

Construction of sustainable composite biofilms from natural biopolymers are greatly promising for alternatives to conventional synthetic polymer packaging, which can not only allow advanced biodegradable packaging applications, such as food and pharmacy, but also alleviate carbon emission and environmental pollution [1]. Starch is a natural biopolymer with advantages of biodegradability, renewability, low cost, good water solubility, and abundant sources, making it a promising biomaterial in food packaging [2,3]. However, pure starch films often suffer from poor barrier properties and low mechanical strength, which significantly limit its packaging applications [4]. To address these issues, a wide range of nanofillers, such as carbon nanotube, graphene, and nanoclay, have been added into a starch matrix to prepare composites with enhanced gas barrier and mechanical properties [5]. However, these nanofillers usually have drawbacks of poor renewability, high cost, limited functionalities, or poor interface interactions. Thus, the development of new renewable nanofillers with various functionalities are urgently needed for high performance sustainable composite biofilms.

Lignin is an abundant component in lignocellulosic biomass and the most abundant aromatic polymer on earth [6]. It is considered an important renewable resource with the potential to replace fossil resources in the production of energy, chemicals, and materials [7]. However, the complex and heterogeneous chemical structure of lignin poses a challenge for its effective utilization [8,9]. In the traditional pulp and paper industry, lignin obtained from lignocellulose biomass is typically burned as industrial waste to support the energy requirements of the pretreatment process [10,11]. This approach fails to realize the potential value of lignin. Usually, the high-value utilization of lignin primarily relies on specific chemical bond breaking and functional group derivatization. For example, lignin with a high content of active hydroxyl groups or C–H on the benzene ring exhibits high reactivity and is desirable for synthesizing polymer materials such as polyurethane and phenolic resin [12]. Preparing phenolic polymers from lignin requires low molecular weight and a high content of β–O–4 ether bonds [13]. Unfortunately, most lignocellulose pretreatment processes lead to condensation reactions between lignin structural units, resulting in the formation of strong C–C bonds and breakage of a large number of β–O–4 bonds; this makes it very difficult to obtain lignin with the desired chemical structure.

Our previous work has shown that two-step liquid hot water (LHW) coupling with deep eutectic solvent (DES) pretreatment is an efficient strategy to achieve the lignocellulose fractionation. Meanwhile the transformation of molecular lignin into lignin nanoparticles (LNPs) is favorable for their high-value utilization [14,15]. Compared with traditional inorganic nanoparticles, LNPs have the advantages of biodegradability, good biocompatibility, ultraviolet resistance, and antibacterial properties, which exhibit great potential in biomedicine, food packaging, microbiological, pharmaceutical, and other fields [16,17]. Owing to the uniform morphology, rich surface chemistry, and the small size, LNPs can be evenly distributed in the polymer matrix with ideal interface compatibility as reinforcement [18,19]. For example, LNPs have been widely incorporated into natural and synthetic polymers, such as polyvinyl alcohol (PVA) [14], bacterial cellulose [20], chitosan [21], polylactic acid [22], polyurethane [23], and high-density polyethylene [24], to prepare multi-functional materials with enhanced mechanical properties. Therefore, incorporating LNPs into a starch matrix is not only of great promise to improve its antioxidant, UV shielding, and mechanical properties for producing a starch-based advanced packaging film, but also provides a high-value utilization approach for lignocellulose biorefineries.

In this paper, we report on sustainable composite biofilms through the incorporation of lignin nanoparticles as the biofiller into a starch matrix towards advanced packaging applications. We selected moso bamboo as the lignocellulose raw material due to its rich sources and low cost. A green and efficient approach including two-step LHW coupling with DES pretreatment and subsequent self-assembly process was employed to prepare LNPs with uniform morphology and size. The starch/LNPs composite films were prepared by the facile solution-casting method. The influence of LNPs on the structure and performance of the composite film was systematically studied. The starch/LNPs composite films demonstrated great potential in soybean oil antioxidation packaging.

## 2. Materials and Methods

### 2.1. Materials

Moso bamboo powder was collected from Chengdu Shuxiang Co., Ltd. (Chengdu, China). Potato starch and 2,2-Diphenyl-1-picrylhydrazyl (DPPH) was purchased from Yuanye Biotechnology Co., Ltd. (Shanghai, China). The spectra/Por® 2 standard RC dry dialysis tube (8–14 kDa) was purchased from Spectrum Laboratories, Inc. (Compton, CA, USA). Choline chloride was purchased from Sigma-Aldrich. (Burlington, MA, USA) Urea, dimethyl sulfoxide (DMSO), HCl solution, KOH solution, and other reagents or solvents were purchased from Kelong Chemical Reagent Co., Ltd. (Chengdu, China).

### 2.2. The Preparation of Lignin Nanoparticles (LNPs)

Lignin was extracted according to our previous work using the liquid hot water (LHW) pre-hydrolysis followed by deep eutectic solvent (DES) extraction [15]. Basically, 50 g moso bamboo powder was soaked in 0.7% sulfuric acid with a solid-to-liquid ratio of 1:15 overnight. Then, the slurry was transferred into an autoclave and reacted at 170 °C for 90 min. After reaction, the solid fraction was separated and rinsed with deionized water until pH was neutral. Then the obtained solid fraction was stored at 4 °C before use. DES used in this study was prepared through mixing urea and choline chloride (molar ratio 2:1) at 80 °C for 30 min with stirring. In total, 10 g of LHW pretreated solid substrate (based on dry weight) was mixed with 200 g of DES in a 500 mL flat-bottomed flask. In order to reduce the viscosity of DES system, 20 g of distilled water was added. After stirring at 100 °C for 3 h, the reaction was cooled to 80 °C. Next, 200 mL of mixed acetone/water (1:1 by volume) solvent was added to decrease the viscosity. Then, 1000 mL distilled water was added into the beaker containing dissolved lignin and DES to precipitate lignin fragments. The precipitated lignin was vacuum-filtered with a nylon film after evaporation of acetone in a fume hood for 12 h. The precipitated lignin was washed with hot deionized water until the pH value reached neutral. The resulting lignin was freeze-dried and stored in a desiccator for further characterization and LNPs preparation.

The prevalent dialysis method was employed to fabricate LNPs using dimethyl sulfoxide (DMSO) as solvent and water as anti-solvent according to our previous procedure [14]. Briefly, 400 mg DES lignin was dissolved in 100 mL DMSO to form a homogeneous solution and then introduced into a dialysis tube. The dialysis process was carried out in tap water. Water was changed every 2 h in the first 12 h and then every 10 h until no DMSO trace was detected in the water. Finally, the obtained LNPs suspension was kept at 4 °C for further characterization.

### 2.3. Preparation of Starch/LNPs Composite Films

Potato starch was dispersed in deionized water to obtain 4% (*w*/*v*) starch solution. Then 30% glycerol (*w*/*w*, on a dry basis of the weight of starch) was added. The solution was heated at about 90 °C for 1 h with 500 r/min magnetic stirrer to accomplish a complete starch gelatinization. LNPs were added at a concentration of 1%, 2%, 3%, 4%, and 5% (*w*/*w*, on a dry basis of the weight of starch) and the resulting dispersions were subjected to further mixing for 2 h, followed by degasification for 1 h using a vacuum pump (−0.09 MPa). Then the solutions were cast on a 200 mm × 100 mm glass plate and dried at 45 °C for 20 h, and then the films were removed from the glass plate and conditioned at 25 °C and 55% relative humidity for 24 h before running further tests. The obtained starch/LNPs (SL) composite films were named based the amount of LNPs added. For example, SL-1 indicates starch composite film mixed with 1 wt% LNPs. The pure starch film without LNPs added was set as blank control. All the biofilms are ~70 um.

### 2.4. Characterizations

The morphologies of LNPs were characterized using a transmission electron microscope (TEM, JEOL JEM-100CX, Akishima, Japan).

The pure starch film and SL composite films were characterized using scanning electron microscopy (SEM, JEOL JSM-5600) and an atomic force microscope (AFM, MultiMode III, Bruker, Bremen, Germany).

Fourier transform infrared spectroscopy (FTIR) was collected with the spectrophotometer (Nicolet 6700, Waltham, MA, USA) in the range of 4000–400 cm^−1^ as the average of 32 scans with a resolution of 4 cm^−1^.

The Philips Analytical X’Pert PRO MPD (Amsterdam, The Netherlands) X-ray diffractometer was employed on the pure starch film and SL composite films to conduct the XRD measurements with an incident Cu-Kα X-ray beam (λ = 1.54 Å). The diffraction patterns were obtained over the 2θ range of 2–60° by steps of 0.02°.

Thermal analysis was performed on a 209F thermos gravimetric analysis (TGA, Netzsch, Selb, German). The films were weighed in alumina pans and heated from room temperature to 800 °C at the heating rate of 10 °C/min under N_2_ atmosphere.

Color parameters including L (lightness), a (red/green), and b (yellow/blue) of SL composite films were determined with an Automatic colorimeter CR-400 (Auyck Optoelectronic Instrument Co., Ltd., Beijing, China). The colorimeter was calibrated using a standard white plate (L = 81.84, a = −0.65, b = 5.55). Color parameters were recorded from three points randomly selected in each SL composite films.

The UV shielding performance and optical transparency of the SL composite films were measured on a UV–2600 UV–Vis spectrophotometer (Shimandzu, Kyoto, Japan) within the scan ranging from 200 to 800 nm.

Mechanical properties of the pure starch film and SL composite films were determined by a universal tensile testing machine with a load cell of 1000 N (Instron-5560, Norwood, MA, USA) at 60 mm min^−1^. The films were cut into a rectangle shape with a length of 80 mm and a width of 10 mm prior to analysis. At least five parallel specimens were tested in each sample group.

Oxygen permeability (OP) of SL composite films was evaluated by deoxidizer absorption method [25]. The centrifuge tube containing a certain amount of deoxidant was sealed with the SL composite films. Then the weight change of the tube was recorded over a period of time in a constant environment (25 °C, RH 50%). OP was calculated as follows:

(1) $$\mathrm{OP}=\frac{\Delta M\;\times \;d}{A\;\times \;t\;\times \;P}$$

where ΔM is the weight change of test tube (g), d is the film thickness (cm), A is the effective area of the film (cm^2^), t is the equilibrium time (s), and P is the oxygen partial pressure (Pa). All films were tested at least three times.

The antioxidant activity of the SL composite films was evaluated by assaying the scavenging of free radical of DPPH [26]. For this, 0.1 g SL composite films was added to each conical flask containing 20 mL ethanol (covered with aluminum foil) with magnetic stirring (150 r/min) at 25 °C for 24 h until dissolved completely. Then a 2 mL 0.01 mM DPPH assay solution was prepared by mixing 1 mL of the film solution. The mixture was incubated in the dark at room temperature for 30 min. In this experiment, ethanol DPPH solution without membrane was used as control. The experiment was repeated three times. The UV absorbance of the mixtures was measured at 517 nm. DPPH scavenging activity was calculated as follows:

(2) $$\mathrm{DPPH}\;\mathrm{radical}\;\mathrm{scavenging}\;\mathrm{activity}\;\left(\%\right)=\frac{A_{\mathrm{DPPH}}-A_{s}}{A_{\mathrm{DPPH}}}\times 100$$

where A_DPPH_ is the absorbance value at 517 nm of the methanol solution of DPPH and A_s_ is the absorbance value at 517 nm of the DPPH assay solution. Tests were performed three times for each specimen and the average values were taken.

### 2.5. Soybean Oil Storage Experiments

The pure starch film, SL-2, and SL-5 were selected for the accelerated oxidation experiments of soybean oil. For this experiment, 150 g of soybean oil was poured into a 250 mL glass bottle, covered with the pure starch film, SL-2, or SL-5 and sealed with rubber bands. The sample without film packaging was used as control. All samples were stored in an oven at 50 °C, RH 25% for 24 days. The peroxidation value (POV), saponification value (SV), and acid value (AV) of soybean oil were measured every 6 days. Tests were performed for each specimen in triplicate and the results averaged.

The POV of the samples was measured according to the method of GB 5009.227-2016 [27]. Briefly, 2 g of extracted soybean oil was weighed in 250 mL iodine flask and 30 mL of acetic acid and trichloromethane mixed solution was added. Then, 1 mL of saturated solution of potassium iodide was incorporated into the measuring bottle and kept for 3 min in a dark place. Subsequently, after addition of 100 mL distilled water to the solution, it was titrated using the standard solution of sodium thiosulfate in the presence of 1 mL starch solution as the indicator, and reached the end point when the blue color disappeared. The POV was calculated according to the following equation:

(3) $$\mathrm{POV}=\frac{\left(v-v_{0}\right)\times c\times 0.1269}{m}\times 100$$

where V is the volume of sodium thiosulfate standard solution (mL) consumed by the sample, V_0_ is the volume of sodium thiosulfate standard solution (mL) consumed in blank determination, c is the concentration of sodium thiosulfate standard solution (mol/L), 0.1269 is the amount of iodine equivalent to 1 mL of sodium thiosulfate standard titration solution, and m is the weight of soybean oil (g).

The SV of the samples was measured according to the method of GB/T 5534-2008 [28]. In this regard, 2 g of soybean oil was weighed in a conical flask and 25 mL of potassium hydroxide and ethanol mixed solution was added. The reflux condensate pipe was connected to the conical flask, and the conical flask was placed on the heating device and constantly shaken to boil it for 1 h. In total, 0.5~1 mL phenolphthalein indicator was added to the mixture and titrated with standard solution of hydrochloric acid. The reaction endpoint was reached when the pink of the indicator disappeared. The SV was calculated according to the following equation:

(4) $$\mathrm{SV}=\frac{\left(v-v_{0}\right)\times c\times 56.1}{m}$$

where V is the volume of hydrochloric acid standard solution (mL) consumed for blank determination, V_0_ volume of hydrochloric acid standard solution consumed by sample determination (mL), c is reagent concentration of standard solution of hydrochloric acid (mol/L), and m is the weight of soybean oil (g).

The acidity is the number of milliliters of potassium hydroxide needed to neutralize the free fatty acids found in 1 g of fat. The AV of the samples was measured according to the method of GB 5009.229-2016 [29]. Usually, a certain weight of soybean oil was dissolved in 100 mL ether-isopropyl alcohol solution. This solution was titrated using potassium hydroxide in the presence of phenolphthalein until a stable reddish color appeared. AV was calculated as the following equation:

(5) $$\mathrm{AV}=\frac{\left(v-v_{0}\right)\times c\times 56.1}{m}$$

where V is volume of standard titration solution used, V_0_ is volume of standard titration solution used for blank determination, c is the concentration of standard titration solution (mol/L), 56.1 is the molar weight of KOH, and m is the weight of the soybean oil analyzed.

## 3. Results

**Scheme 1:**
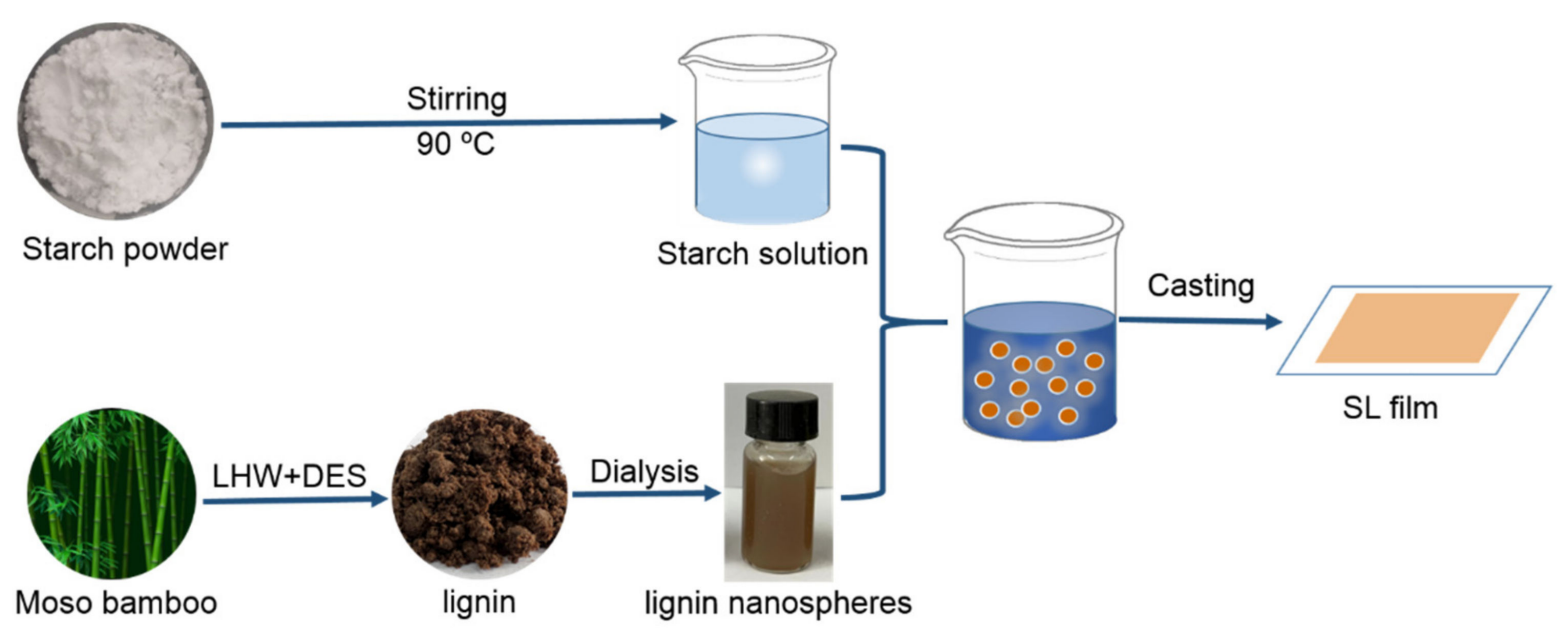
Schematic illustrating of fabrication process of Starch/LNPs composite film.

The detailed procedure to fabricate the starch/LNPs (SL) composite films is shown in Scheme 1. Our previous reports [14,15] demonstrated that the two-step pretreatment of LHW and DES is a feasible approach to realize the lignocellulose fractionation. To achieve full utilization of lignocellulose, a two-step pretreatment strategy, consisting of mild LHW and DES extraction, has been applied to produce high-purity lignin components while facilitating the conversion of cellulose/hemicellulose components to a hexose/pentose sugar platform [14,15]. The obtained DES lignin could be used to prepare LNPs via the self-assembly process driven by the π-π stacking interactions when anti-solvent is introduced into the lignin solution. During the self-assembly process, the hydrophobic aromatic skeletons of lignin aggregate in the anti-solvent to form the particle core, while the aliphatic hydroxyl and hydrophilic phenolic groups form the particle shell.

### 3.1. Preparation and Characterization of Lignin Nanoparticles

**Figure 1:**
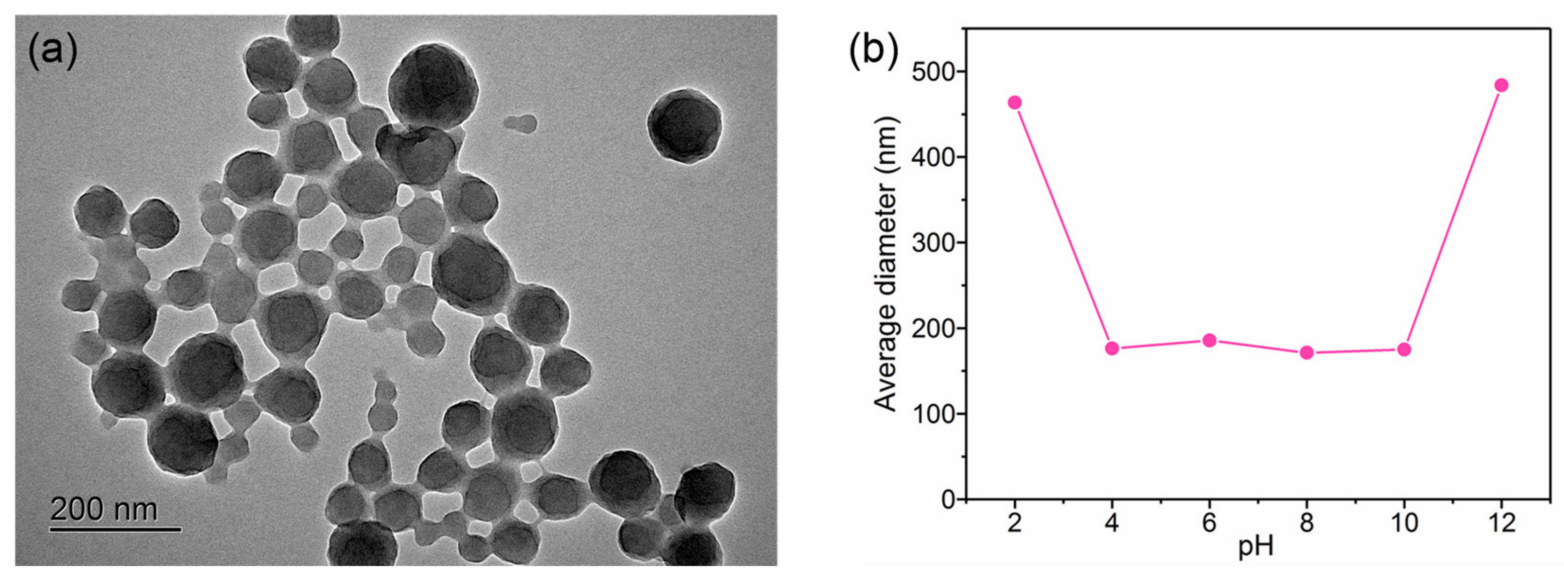
(**a**) TEM and (**b**) effect of pH on average hydrodynamic diameter of lignin nanoparticles.

The core–shell structure and well-defined spherical shape of the LNPs prepared from moso bamboo were confirmed using TEM (Figure 1a); particle sizes of moso bamboo LNPs were in the range of 30~130 nm, and the shell thickness was about 5~10 nm. The effect of pH on average hydrodynamic diameters of LNPs dispersion were further analyzed using dynamic light scattering (DLS) (Figure 1b). It was shown that particle sizes of LNPs were similar or slightly higher than what is shown in their TEM images, indicating slight aggregations among particles. The particle sizes became larger at low pH < 4 and high pH > 10 due to LNPs’ agglomeration and disassembly, respectively [15]. The regular-sphere structure, uniform particle size, and high stability in a wide pH range of LNPs are favorable as the green nanofillers for the production of starch nanocomposite films.

### 3.2. Structure and Characterizations of Starch/Lignin Nanoparticle Composite Films

**Figure 2:**
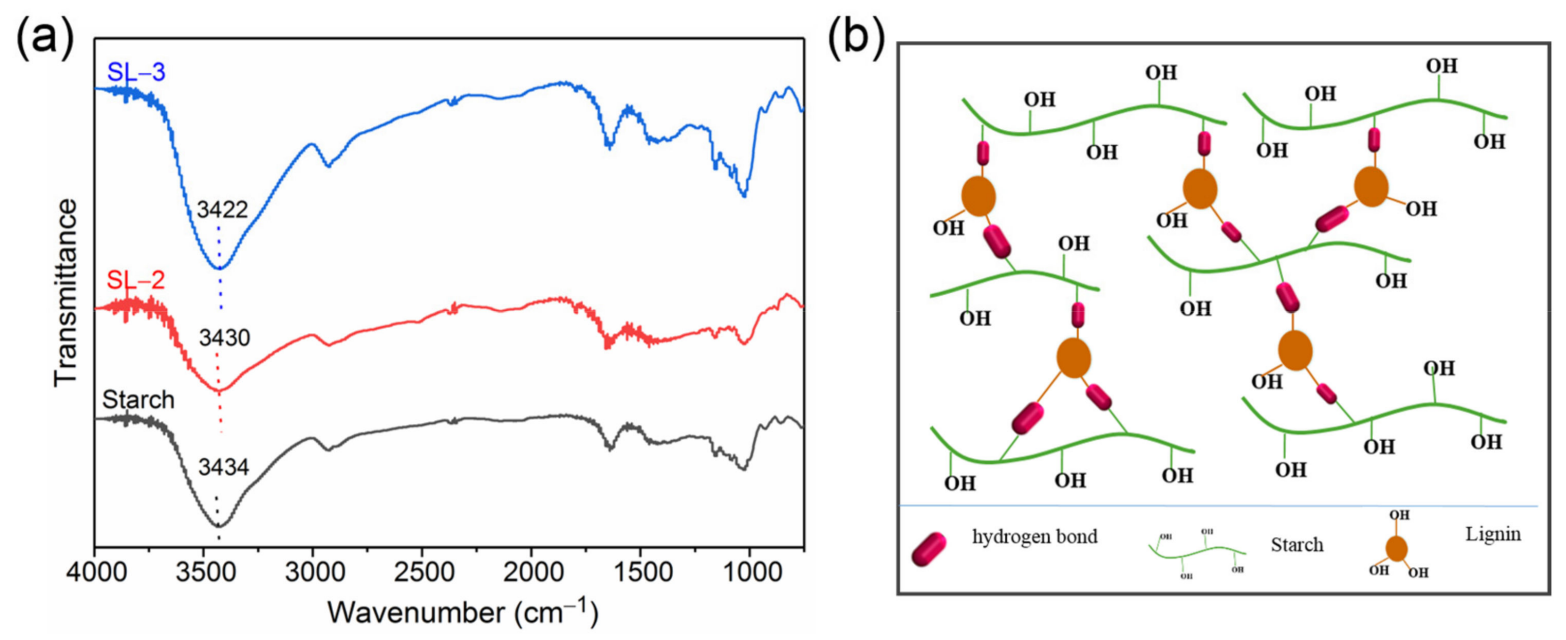
FTIR spectra (**a**) of starch film and SL composite films incorporating 2% and 3% LNPs, and schematic illustration (**b**) of the interaction between starch matrix and LNPs.

Figure 2 shows the Fourier transform infrared spectroscopy (FTIR) spectra of pure starch films and SL composite films with different contents of LNPs. As shown in Figure 2a, the characteristic absorption peaks of starch films are located at 3434 cm^−1^, 2920 cm^−1^, and 1640 cm^−1^, corresponding to the stretching vibration of O–H, the stretching vibration of C–H bond, and the bending vibration of –OH, respectively [30]. The broad peak at around 3434 cm^−1^ is related to the formation of inter- and intra-molecular hydrogen bonds by hydroxyl groups [31]. With addition of LNPs, the intensity and position of the above-mentioned characteristic peaks of the composite film changed slightly. With 2% and 3% LNPs added, the characteristic peak at 3434 cm^−1^ moved to 3430 cm^−1^ and 3422 cm^−1^, respectively, which indicates that there is a stronger hydrogen bonding interaction between LNPs and starch molecules [32]. As shown in Figure 2b, LNPs’ surfaces possess abundant hydroxyl groups, which can act as crosslinking sites to connect with neighboring starch molecular chains through the formation of hydrogen bonding [33]. These hierarchical dynamic interfacial interactions greatly benefit the enhancement of the mechanical and functional properties.

**Figure 3:**
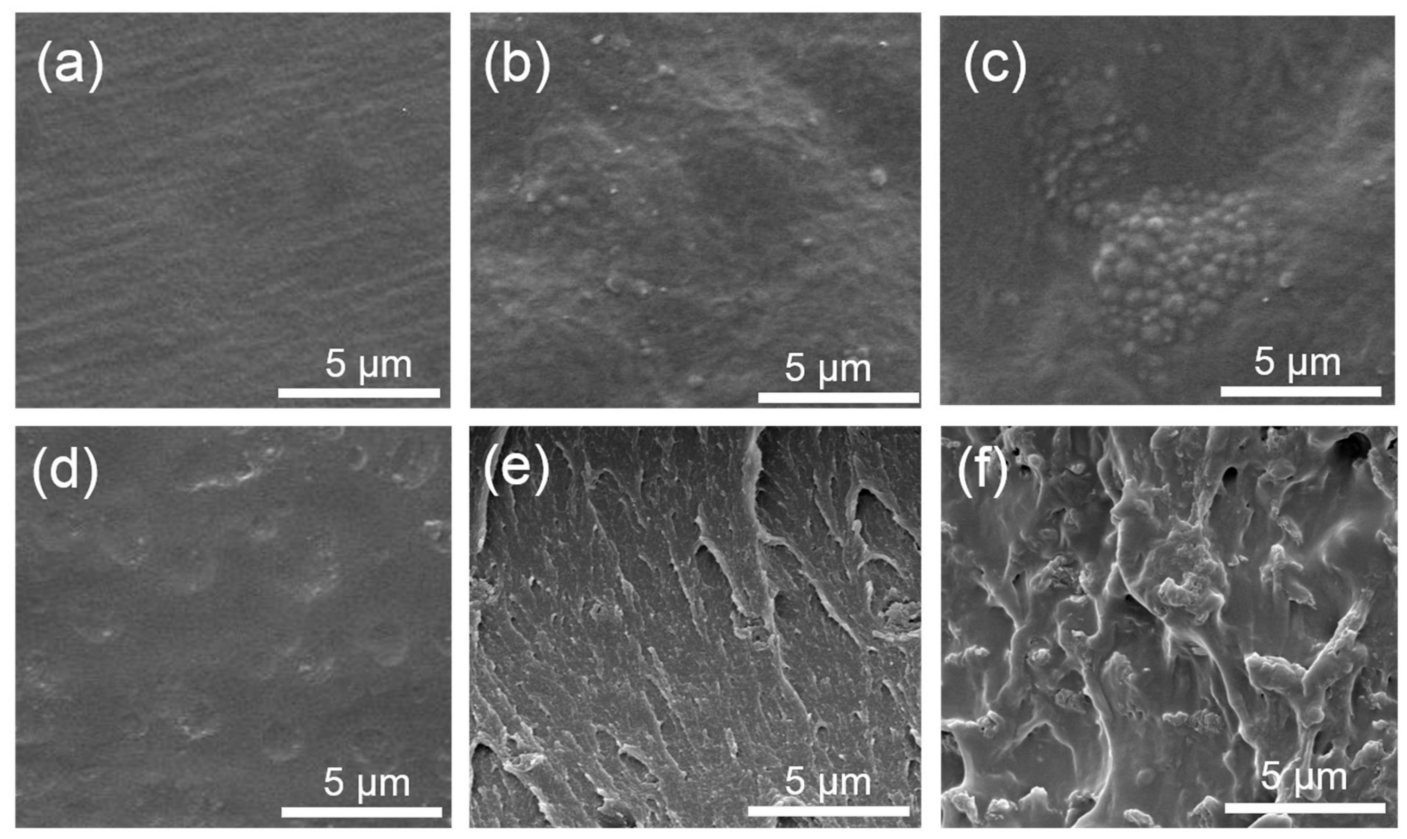
SEM images of surface and cross section of starch film (**a**,**d**) and SL composite films containing 2% (**b**,**e**) and 5% (**c**,**f**) LNPs.

To gain insight of the distribution of LNPs in a starch matrix, we conducted the morphology characterization of composites films. Figure 3a,d shows the SEM images of the surface and cross-section of the pure starch film, which exhibits a uniform and smooth microscopic morphology. Upon addition of 2% LNPs, the surface and cross-section of the SL-2 became slightly rough, but the LNPs were uniformly distributed within the starch film matrix. However, increasing the LNP content to 5% resulted in partial particle agglomeration and a rougher surface, with discontinuous cross-sections of the composite film. This may be attributed to the large amount of LNPs with hydroxyl groups which could easily form hydrogen bonds between molecules, resulting in particle aggregations and uneven dispersion within the matrix [34].

**Figure 4:**
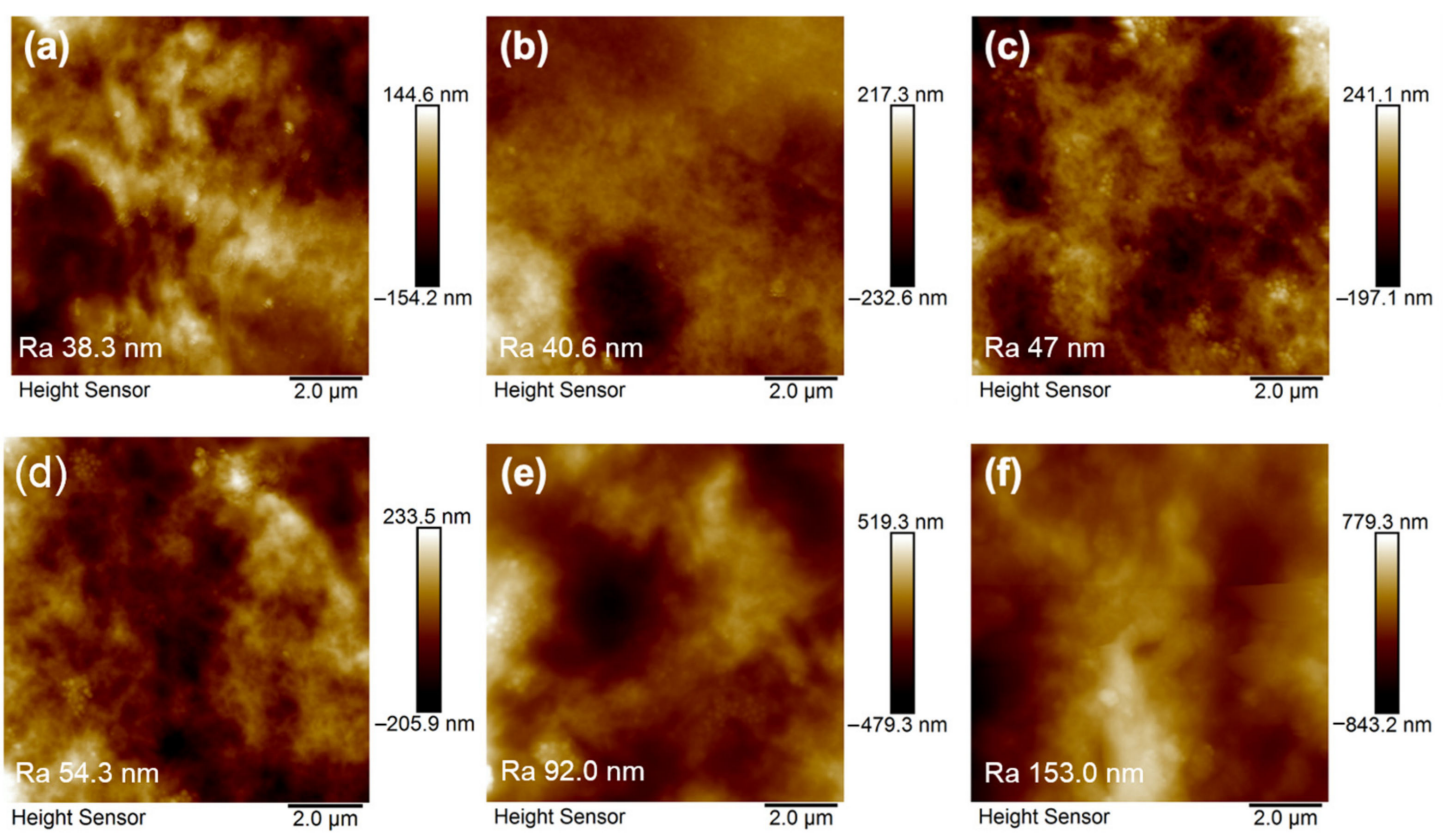
AFM images of starch film (**a**) and SL composite films (**b**–**f**) containing 1%, 2%, 3%, 4%, and 5% LNPs.

Additionally, the surface roughness of the SL composite films was measured using AFM (Figure 4). The results show that the average surface roughness (Ra) of the SL composite films increased from 38.3 nm to 153 nm as the amount of LNPs increased from 0% to 5%. This increase in roughness was more significant when the LNP content exceeded 3%, consistent with the SEM results. These microscopic morphology changes suggest that high LNP content can disrupt the continuity of the starch matrix, which may adversely affect the mechanical properties of the resulting composite film. Therefore, it is necessary to optimize the amount of LNPs addition to achieve the desired properties of the composite films.

**Figure 5:**
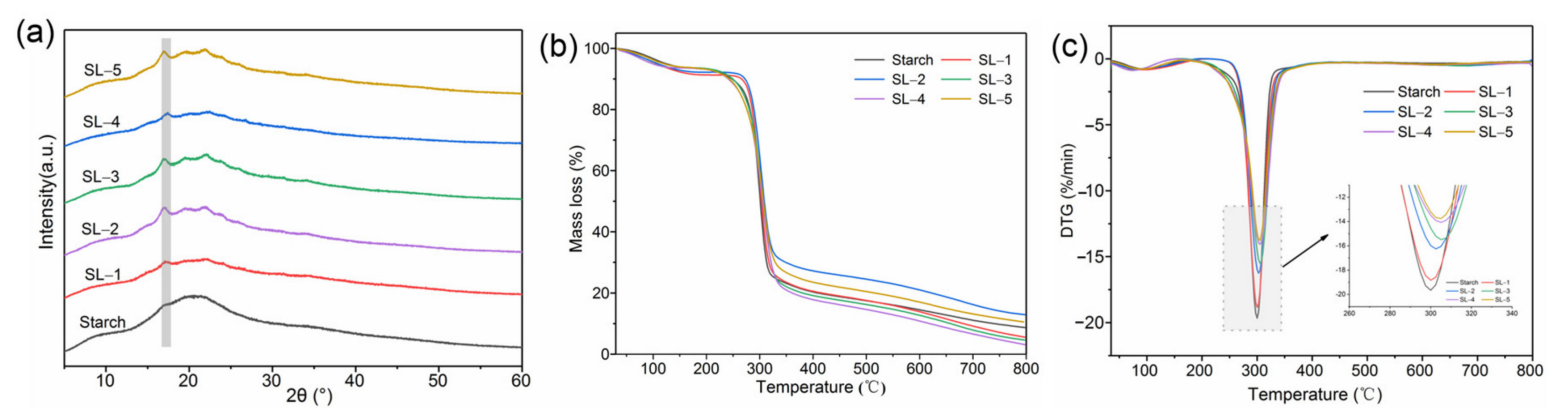
XRD pattern (**a**), TG (**b**) and DTG (**c**) curves of starch film and SL composite films containing 1% to 5% LNPs.

Figure 5a illustrates the X-ray diffraction patterns of the pure starch film and SL composite films with varying amounts of LNPs. The pure starch film exhibits 2 small crystallization peaks at 17 ° and 23 °, which indicates the presence of B-type crystalline [35]. Upon adding LNPs, the diffraction peaks of the SL composite films become sharper and their intensity significantly increases, particularly at 17°, indicating that the addition of LNPs promoted the formation of the starch B-type crystalline. This phenomenon may be attributed to the nucleation effect of LNPs, which promotes the crystal formation and facilitates the growth process of starch [17,36].

The thermal stabilities of the pure starch film and SL composite films with varying LNP contents are illustrated in Figure 5b,c. The TGA curves of all samples display two distinct weight loss stages. The first stage appears at the temperature range of 30–150 °C, corresponding to the evaporation loss of water. The second stage ranging from 150 to 350 °C represents the primary weight loss stage of the samples, which is attributed to the thermal decomposition of starch and lignin. Figure 5c demonstrates that the addition of LNPs elevates the maximum thermal decomposition temperature of the SL composite films (~5–8 °C higher than the pure starch film) indicating an improvement in thermal stability. This improvement is primarily attributed to the strong interaction and good compatibility between LNPs and the starch matrix. Additionally, the presence of nanoparticles forms a barrier for the thermal decomposition of the polymer, leading to an increase in the decomposition temperature [14]. Luo et al. [37] also reported similar findings that the addition of LNPs hindered the thermal decomposition of polyvinyl alcohol, resulting in an increase in its degradation temperature.

### 3.3. Optical and UV Shielding Properties of Starch/Lignin Nanoparticle Composite Films

**Figure 6:**
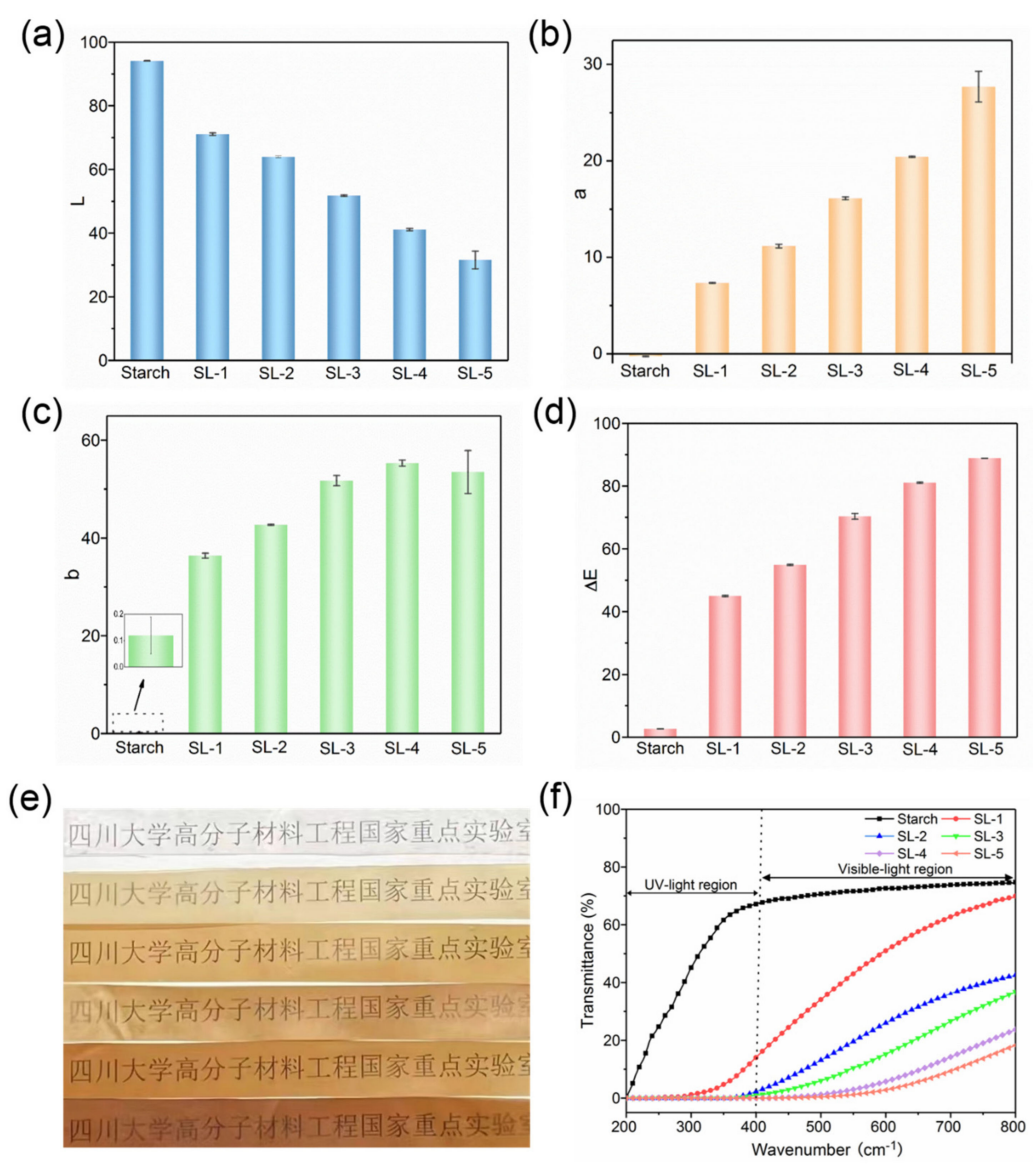
Color index: (**a**) L (lightness), (**b**) a (red/green) value, (**c**) b (yellow/blue) value, and (**d**) △E (total color difference), and (**e**) digital photographs (the visibility of the chinese characters (Sichuan University State Key Laboratory of Polymer Materials Engineering) under the films.) and (**f**) UV-vis light transmittance spectra of starch film and SL composite films containing 1% to 5% LNPs.

Next, we systematically studied the optical properties of the as-prepared films. Figure 6a shows that the addition of LNPs causes a gradual decrease in the L (lightness) value of the SL composite films when compared to the pure starch film. Additionally, the a (red/green) value (Figure 6b), b (yellow/blue) value (Figure 6c), and total color difference (△E) value (Figure 6d) increase, indicating a decrease in brightness and a noticeable shift towards reddish or yellowish hues. This color shift is mainly attributed to the color of the LNPs themselves. Nieto et al. [38] observed similar results when adding colored curcumin nanoparticles to banana starch film.

The digital photo of the SL composite films in Figure 6e demonstrates that the pure starch film is colorless and transparent, while the SL composite films are brown. Their color intensity increases with the increase in LNP content. This result is consistent with the color index change results. It is worth noting that despite a slight decrease in light transmittance in the visible light region (400–800 nm) after the addition of LNPs, the SL composite films exhibit excellent UV shielding properties (Figure 6f). The pure starch film displays no absorption or blocking of ultraviolet light due to its colorless and transparent nature. Even with only a 1% addition of LNPs, the SL-1 demonstrates a substantial shielding effect on ultraviolet light, while maintaining high visible light transmittance efficiency. At the 2% addition of LNPs, SL-2 achieves nearly complete UV shielding. This is primarily attributed to the phenolic hydroxyl group in the LNPs structure, as well as the carbonyl group of the phenyl propane side chain, and the conjugated double bond of the benzene ring, all of which possess strong absorption properties towards ultraviolet light. The phenolic hydroxyl groups could shield UV light by absorbing its photon energy and then converting it to heat, which is further gradually released into the environment without causing the photo-catalytic degradation of the starch matrix; this is in contrast to metal oxides [17,39]. Furthermore, the presence of LNPs results in a strong light scattering effect, causing the decrease in light transmittance of the SL composite films. These results suggest that the appropriate addition of LNPs to the starch film can largely enhance its UV shielding properties without significantly affecting its visible light transparency, thus providing good practical value for its application in food packaging fields [40].

### 3.4. Mechanical Properties of Starch/Lignin Nanoparticle Composite Films

**Figure 7:**
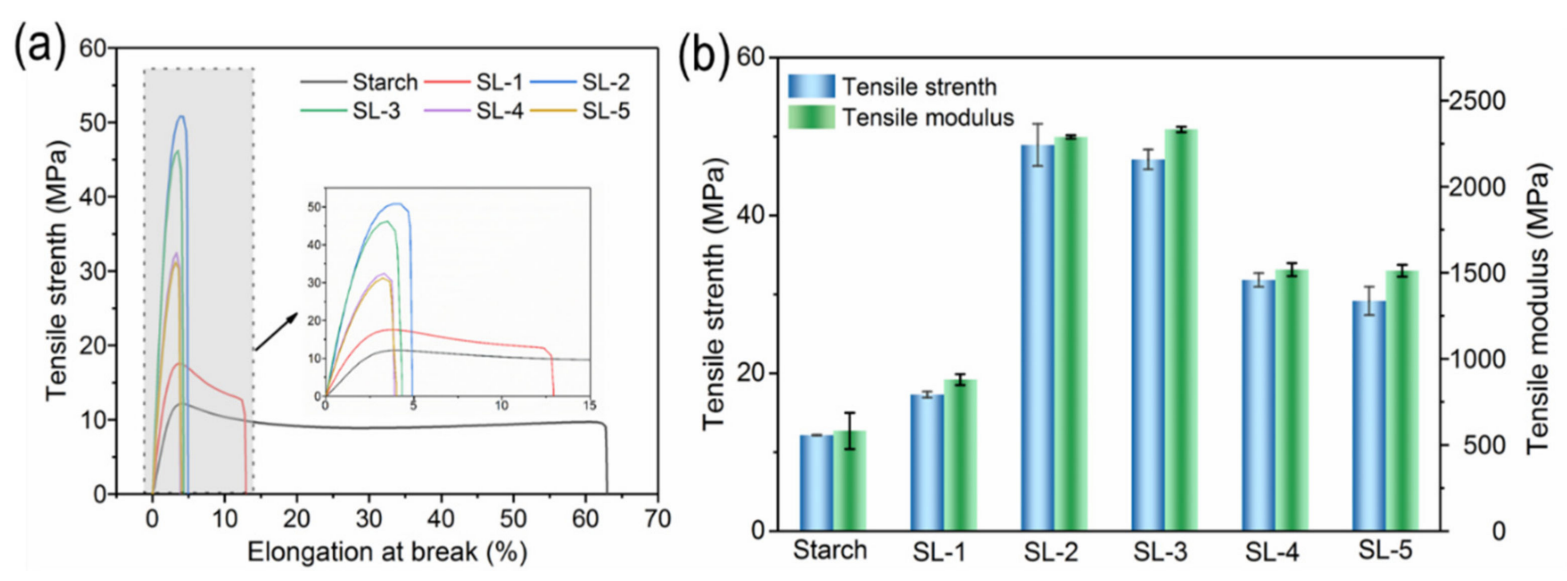
(**a**) Stress–strain curves and (**b**) Tensile strength and modulus of starch film and SL composite films containing 1% to 5% LNPs.

Figure 7 depicts the stress–strain curves of the SL composite films at varying LNP contents. The pure starch film exhibits poor mechanical properties, with a tensile strength of 12.1 MPa and an elastic modulus of 581.8 MPa, which greatly limits its application as a food packaging material. However, the addition of LNPs significantly improves the tensile strength and elastic modulus of the composite film, reaching its highest value when the content of LNPs is 2%~3%. For instance, the SL-2 film has a tensile strength of 48.9 MPa and an elastic modulus of 2288.8 MPa, which is 3.04 times and 2.93 times higher than those of the pure starch film, respectively. The reinforcement effect is attributed to the distribution of LNPs in the starch matrix, forming hydrogen bonds that have good compatibility and physical reinforcement with starch [41,42]. However, a high content of LNPs causes particle agglomeration (Figure 7), adversely affecting the mechanical properties of the composite membrane and partially offsetting the reinforcement effect, reducing the material strength. Furthermore, the formation of a stable network structure between starch molecules and LNPs reduces the mobility of starch macromolecular chains, leading to a decrease in the toughness of the composite film and lower elongation at break.

### 3.5. Antioxidant Properties of Starch/Lignin Nanoparticle Composite Films

**Figure 8:**
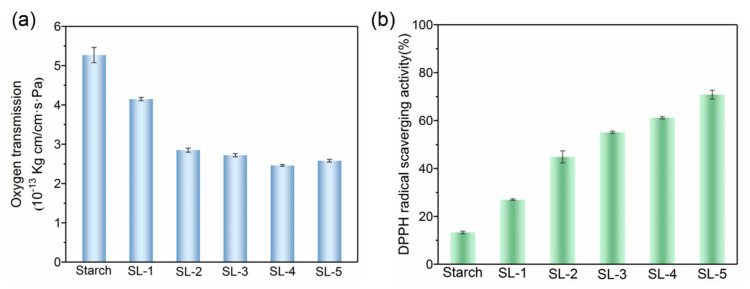
(**a**) Oxygen permeability and (**b**) DPPH radical scavenging rate of starch film and SL composite films containing 1% to 5% LNPs.

The oxygen transmission rate (OP) is a fundamental parameter to assess the gas barrier properties of food packaging films [25]. As illustrated in Figure 8a, The OP value of the pure starch film was 5.3 × 10^−13^ kg cm⋅cm^−2^⋅s^−1^⋅Pa^−1^, while the OP of SL-2 was significantly reduced to 2.85 × 10^−13^ kg cm⋅cm^−2^⋅s^−1^⋅Pa^−1^. The OP of the SL composite films significantly decreased as the LNP content increased in comparison with the pure starch film. This is attributed to the LNPs acting as a barrier to oxygen permeation and increasing the tortuosity of the oxygen diffusion path within the membrane, preventing oxygen consumption and absorption by the deoxidizer. As a result, the oxygen transmission rate of the SL composite films decreased [43,44]. Wu et al. [33] conducted similar tests on the starch/gelatin composite film with resveratrol and obtained comparable results.

Furthermore, the antioxidant performance of the composite films was evaluated by measuring the DPPH radical scavenging activity, as shown in Figure 8b. The DPPH of pure starch film was 13.3%, while that of SL-2 and SL-5 composite films increased to 44.8% and 70.8%, respectively. The results demonstrated that the antioxidant activity of the SL composite film gradually increased with the addition of LNPs. This is due to the active phenolic hydroxyl groups present in the LNPs that scavenge free radicals through an electron transfer process, thus endowing the SL composite films with enhanced antioxidant properties [14]. Therefore, introducing LNPs to the starch film can provide it with good antioxidant properties, making it a promising material for food packaging applications that require antioxidation properties.

### 3.6. Potential Application for Oil Packaging

As the edible component of lignocellulose, lignin has been used for food packaging films recently. Unlike other metal oxide or mineral fillers that usually exhibited environmental and health risks, lignin nanofillers were much biocompatible. Therefore, the health risk issue could be limited even if it migrated into food when used for food packaging. To further explore the food packing application of SL composites films, we evaluated the effect of composite films on delaying oxidative deterioration of soybean oil. Peroxide value (POV), saponification value (SV), and acid value (AV) are important indicators for evaluating the quality of oils and fats. POV indicates the degree of oxidation, with a higher value indicating more serious oxidation [45]. SV indicates the molecular weight of fatty acids in oils, with a higher value suggesting a higher degree of rancidity [46]. AV reflects the content of free acid in fat produced by the oxidative hydrolysis of triglyceride [46].

**Figure 9:**
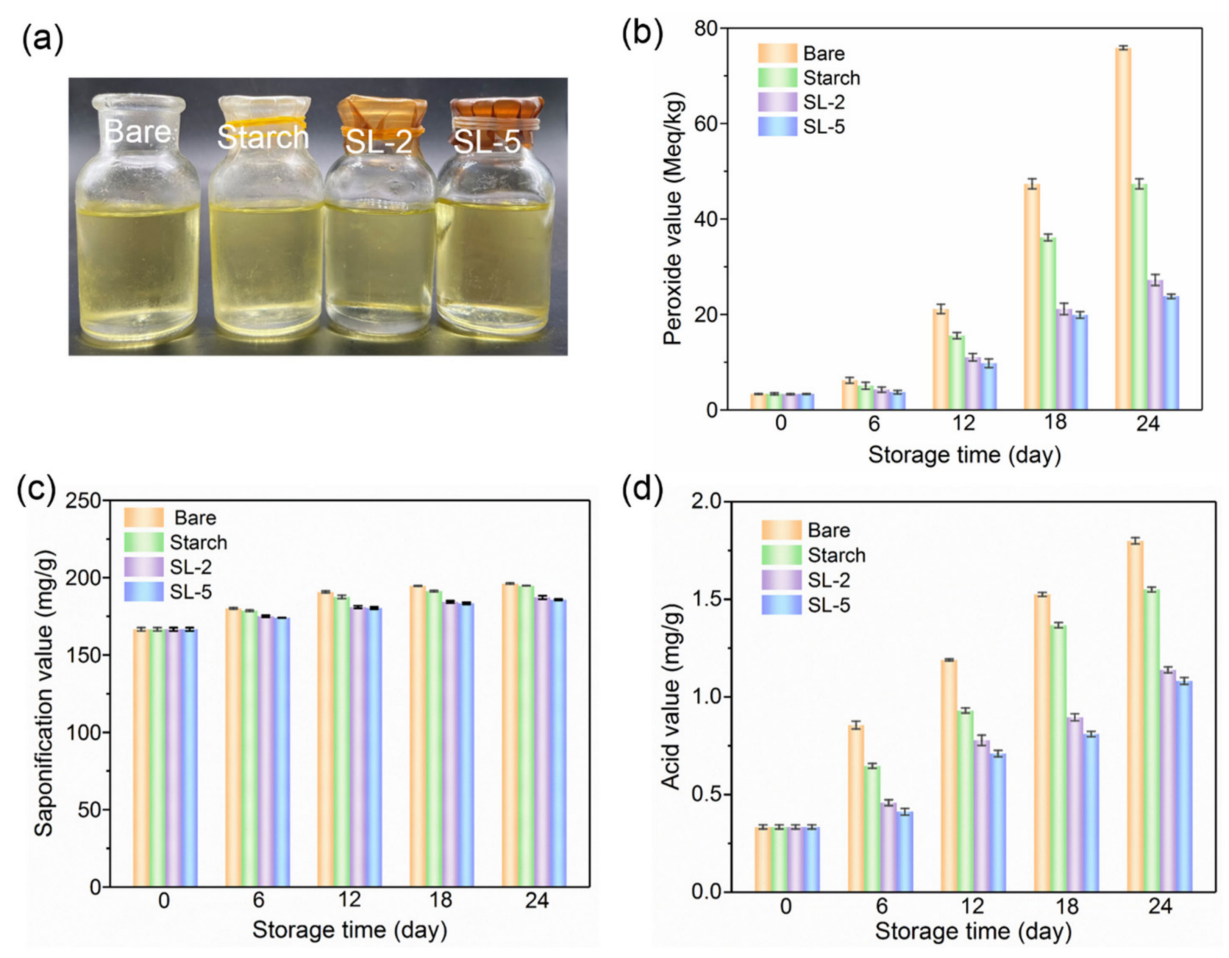
(**a**) Image of experiment for soybean oil accelerated oxidation, changes of (**b**) the peroxide value, (**c**) saponification value, (**d**) acid value of peanut oil packaged by starch film, SL-2 and SL-5 composite films.

As shown in Figure 9, with the prolongation of storage time, the POV, SV, and AV of all oil samples increased to varying degrees, indicating obvious oxidative deterioration of soybean oil under high temperature storage. Notably, the unsealed control sample showed the fastest increase in these indicators, while the pure starch film, SL-2, and SL-5 composite film sealing treatments showed a decreasing trend in order. On the 24th day of storage, the control oil sample had the highest POV, SV, and AV of 75.9 meq/kg, 196.2 mg/g, and 1.80 mg/g, respectively, because the soybean oil sample was directly exposed to external oxygen. Whereas, these indexes of the packaged samples were 47.4 meq/kg, 194.8 mg/g, 1.55 mg/g and 75.9 meq/kg, 196.2 mg/g, 1.80 mg/g for the pure starch film and SL-2 composite film, respectively. The SL composite films were observed to have a better effect on delaying the oxidation of soybean oil. However, when LNP content increased to 5%, partial particle agglomeration appeared in the SL-5 composite film, which could be observed from SEM (Figure 3) and AFM (Figure 4). Therefore, there was little change on delaying oxidative deterioration of soybean oil packaged with SL-2 and SL-5. Lignin is an amphiphilic polymer, when it was used for LNP production in this work, its hydrophobic aromatics were aggregated to form the core, while its hydrophilic hydroxyl groups were aggregated to form the shell. This well fabricated core–shell structure endowed LNPs with considerable UV blocking and antioxidant properties. Basically, LNPs block the UV light by absorbing its photon energy and further converting it to heat with the corresponding hydrophilic chromophore (mainly phenolic hydroxyl groups) exposed on the LNP shell. Then, the generated heat was gradually released out of the nanocomposite films. The chromophore could also easily quench active radicals generated by oxygen oxidation through an electron transfer process. Therefore, there were excellent oxygen barrier properties and a higher antioxidant activity of the SL composite films (as shown in Figure 8), which effectively prevented or reduced oxygen from entering the oil bottle and interrupting the oxidation reaction process of soybean oil [47]. The excellent UV resistance of SL composite films (Figure 6f) was also beneficial in slowing down the oxidation induced by ultraviolet light in practical applications [48].

Similar research results have been reported in grapefruit-peel-based composite films with added tea polyphenols [47], gelatin/pectin composite films with added resveratrol [49], and starch/gelatin composite films [33]. Overall, our starch-based composite film functionalized using LNPs can be utilized as an excellent antioxidation food packaging material.

## 4. Conclusions

In this work, starch-lignin nanoparticles (SL) composite films were developed via the conventional solution-casting method. Their structural, thermal, optical, UV shielding, mechanical, and antioxidant properties were systematically characterized. FTIR and morphological analysis confirmed the formation of inter-molecular hydrogen bonding interactions between starch and LNPs. The SL-2 composite film presents the highest tensile strength of 48.9 MPa and an elastic modulus of 2288.8 MPa, which is 3.04 times and 2.93 times higher than those of the pure starch film, respectively. LNPs also improved the thermal stability of SL composite films by ~5–8 °C due to their good compatibility with pure starch. Meanwhile, the addition of LNPs can provide excellent UV shielding performance. LNPs added to the starch matrix increases the oxygen permeation diffusion path, reducing the oxygen transmission rate, and endows the SL composite films with good DPPH free radical scavenging antioxidant activity. In addition, SL composite films could significantly slow down the oxidative deterioration of soybean oil. This work provided the appropriate formula for developing SL composite films with desirable properties that are promising for use in food preservation and the sustainable packaging industry.

## Data Availability

The data presented in this study are available on request from the corresponding author. The data are not publicly available due to privacy.
